# Rare variants in complement system genes associate with endothelial damage after pediatric allogeneic hematopoietic stem cell transplantation

**DOI:** 10.3389/fimmu.2023.1249958

**Published:** 2023-09-13

**Authors:** Lilli Leimi, Jessica R. Koski, Outi Kilpivaara, Kim Vettenranta, A. Inkeri Lokki, Seppo Meri

**Affiliations:** ^1^ Pediatric Research Center, Children’s Hospital, Helsinki University Hospital, University of Helsinki, Helsinki, Finland; ^2^ Applied Tumor Genomics Research Program, Faculty of Medicine, University of Helsinki, Helsinki, Finland; ^3^ Department of Medical and Clinical Genetics, Faculty of Medicine, University of Helsinki, Helsinki, Finland; ^4^ Medicum, Faculty of Medicine, University of Helsinki, Helsinki, Finland; ^5^ Diagnostic Center, Helsinki University Hospital, Helsinki, Finland; ^6^ Department of Bacteriology and Immunology and Translational Immunology Research Program, University of Helsinki, Helsinki, Finland

**Keywords:** hematopoietic stem cell transplantation, hematopoietic stem cell transplantation adverse effects, acute toxicity, vascular complications, complement system, endothelial damage

## Abstract

**Introduction:**

Complement system has a postulated role in endothelial problems after hematopoietic stem cell transplantation (HSCT). In this retrospective, singlecenter study we studied genetic complement system variants in patients with documented endotheliopathy. In our previous study among pediatric patients with an allogeneic HSCT (2001-2013) at the Helsinki University Children´s Hospital, Finland, we identified a total of 19/122 (15.6%) patients with vascular complications, fulfilling the criteria of capillary leak syndrome (CLS), venoocclusive disease/sinusoidal obstruction syndrome (VOD/SOS) or thrombotic microangiopathy (TMA).

**Methods:**

We performed whole exome sequencing (WES) on 109 patients having an adequate pre-transplantation DNA for the analysis to define possible variations and mutations potentially predisposing to functional abnormalities of the complement system. In our data analysis, we focused on 41 genes coding for complement components.

**Results:**

50 patients (45.9%) had one or several, nonsynonymous, rare germline variants in complement genes. 21/66 (31.8%) of the variants were in the terminal pathway. Patients with endotheliopathy had variants in different complement genes: in the terminal pathway (C6 and C9), lectin pathway (MASP1) and receptor ITGAM (CD11b, part of CR3). Four had the same rare missense variant (rs183125896; Thr279Ala) in the C9 gene. Two of these patients were diagnosed with endotheliopathy and one with capillary leak syndrome-like problems. The C9 variant Thr279Ala has no previously known disease associations and is classified by the ACMG guidelines as a variant of uncertain significance (VUS). We conducted a gene burden test with gnomAD Finnish (fin) as the reference population. Complement gene variants seen in our patient population were investigated and Total Frequency Testing (TFT) was used for execution of burden tests. The gene variants seen in our patients with endotheliopathy were all significantly (FDR < 0.05) enriched compared to gnomAD. Overall, 14/25 genes coding for components of the complement system had an increased burden of missense variants among the patients when compared to the gnomAD Finnish population (N=10 816).

**Discussion:**

Injury to the vascular endothelium is relatively common after HSCT with different phenotypic appearances suggesting yet unidentified underlying mechanisms. Variants in complement components may be related to endotheliopathy and poor prognosis in these patients.

## 1 Introduction

Allogeneic hematopoietic stem cell transplantation (allo-HSCT) is a well-established therapy for hematologic and lymphoid malignancies, but also for disorders of the immune or hematopoietic systems and metabolism incurable by other treatments. Despite the improvement in prognosis, toxicity and treatment-related mortality (TRM) still remain challenges (1, 2). In our recent study (3) nearly half (56/122, 45.9%) of the pediatric patients, who underwent allo-HSCT, had at least one severe adverse event (grade 3 or 4 by the Common Terminology Criteria for Adverse Events, CTCAE, 4.03 classification). Endothelial cell activation and dysfunction have recently been indicated in many severe complications of allo-HSCT (4). Thrombotic microangiopathy (TMA) is one of the best known endotheliopathy syndromes and a relatively common complication after HSCT affecting 10-20% of the patients (5–8). Previously, we identified a total of 19/122 (15.6%) patients with vascular complications, fulfilling the criteria of capillary leak syndrome (CLS), veno-occlusive disease/sinusoidal obstruction syndrome (VOD/SOS) or TMA. The patients with endotheliopathy had a poorer 5-year overall survival than those without it (77% versus 26%, p<0.001) (3).

Endothelial cells can be damaged and activated during the HSCT process by several mechanisms, including previous chemotherapy, conditioning (e.g. irradiation), drugs used in the transplantation process (such as G-CSF or calcineurin inhibitors), cytokines produced by activated immune cells or the injured tissues, infections, engraftment and alloreactivity (9–11). Injury and inflammation involve neutrophil activation leading to formation of neutrophil extracellular traps (NETs). NETs and other released intracellular materials are thought to activate the complement system (12, 13). On the other hand, overactive or activated complement system has been shown to disturb the normal function of the endothelium in many disease states (14–18). Previously, endotheliopathy in HSCT was considered to follow the “two-hit” –hypothesis. First, many risk factors in the conventional chemo or conditioning phase make the endothelium more vulnerable and procoagulant (Hit 1). This is followed by one or more factors (medications, infections, possible antibodies, or alloreactivity) in the post-HSCT recovery phase (Hit 2) promoting endothelial injury and related problems (19). Although not emphasized earlier, the second hit could, in addition to procoagulant activity, also involve “procomplement” activity with changes in the ability of endothelial cells to protect themselves against complement attack (5). Currently a “three hit” –hypothesis, where the first hit is the patients´ genetic predisposition to endothelial complications, is considered most relevant to the disease pathogenesis (5, 11, 20, 21).

Problems characterized by endothelial dysfunction vary in their clinical phenotype. Still, all of these share a common denominator: endothelial injury accompanied by proinflammatory, prothrombotic and proapoptotic processes likely related to complement activation (4, 8, 10). Here we focused on the following three entities: capillary leak syndrome (CLS), HSCT-related thrombotic microangiopathy (HSCT-TMA) and veno-occlusive disease/sinusoidal obstruction syndrome (VOD/SOS). Other endotheliopathy syndromes, such as engraftment syndrome (ES), idiopathic pneumonia syndrome (IPS), posterior reversible encephalopathy syndrome (PRES), peri-engraftment respiratory distress syndrome (PERDS) and refractory acute GVHD will not be addressed here.

In this retrospective study, because of the postulated role of complement, we studied genetic complement system variants in patients with documented endotheliopathy. Thereby, our aim was to lay basis for understanding potential mechanisms behind the acute complications seen post-HSCT.

## 2 Materials and methods

### 2.1 Patients

Patient selection was accomplished as previously described (3). We studied a cohort of 109 pediatric patients, who underwent allo-HSCT between 1/2001 and 12/2013 at the Helsinki University Children´s Hospital, Finland, and had an adequate pre-transplantation DNA available for whole exome sequencing (WES) and further bioinformatics and statistical analysis. The key demographic and clinical data of the endotheliopathy patients versus others are given in **Table 1**. All patients were of Finnish ancestry.

**Table 1:** The demographics of the patients with endotheliopathy and those without having received allo-HSCT at the Helsinki University Children´s Hospital during 2001-2013.

Characteristics	Endotheliopathy	Others	p-value
Number of patients, n	19	103	
Malignant/non-malignant, n (%)	16 (84.2)/3 (15.8)	82 (79.6)/21 (20.4)	0.763
Male/female, n (%)	8 (42.1)/11 (57.9)	68 (66.0)/35 (34.0)	0.070
*Diagnosis, n (%)*			0.282
ALL/NHL	15 (78.9)	58 (56.3)	
AML/CML	1 (5.3)	21 (20.4)	
SAA/FA/MDS/JMML/other hematopoietic^1^	2 (10.5)	16 (15.5)	
Immunological and metabolic disorders	1 (5.3)	8 (7.8)	
*Donor type, n (%)*			0.053
Sibling	3 (15.8)	48 (46.6)	
MUD	15 (78.9)	47 (45.6)	
Cord blood	1 (5.3)	5 (4.9)	
Family/haplo	0 (0.0)	3 (2.9)	
fTBI, yes/no, n (%)	14 (73.3)/5 (26.3)	84 (81.6)/19 (18.4)	0.529
Relapse after HSCT, yes/no, n (%)	3 (15.8)/16 (84.2)	21 (20.4)/82 (79.6)	0.763
Overall survival, n (%)	5 (26.3%)	79 (76.7)	<0.001
Cause of death, relapse/toxicity, n (%)	2 (10.5)/12 (63.2)	19 (18.4)/5 (4.9)	<0.001
Number of DNA-samples, n (%)	17 (89.5)	92 (89.3)	
*Number of patients with observed complement gene variants, n (%)*			0.408
No variant	12 (70.5)	47 (51.1)	
One variant	4 (23.5)	26 (28.3)	
Two different variants	1 (5.9)	16 (17.4)	
More than two different variants	0 (0.0)	3 (3.3)	

1 No sickle cell disease patients included.

HSCT, hematopoietic stem cell transplantation; MUD, matched unrelated donor; fTBI, fractioned total body irradiation.

The study was approved by the Research Ethics Committee of the Helsinki University Hospital (79/13/03/03/2016, update 3082/2018). All samples and data were obtained following authorization by the ethics committee and the national supervisory authority Valvira. Written, informed consents were not attainable due to the retrospective nature of the study also including deceased patients.

### 2.2 Outcome measures

DNA analysis aimed to define possible variations and mutations in classical, lectin or terminal pathway factors or in the membrane-bound components of the complement system, potentially predisposing to activation or dysfunction of the complement cascade.

### 2.3 Data collection

Genomic DNA was isolated by the Finnish Red Cross Blood Service (HLA-lab), Helsinki, Finland, from pre-transplant peripheral blood samples. DNA-samples were collected from both the recipient and donor for HLA-matching. Clinical data was collected retrospectively from the medical records focusing on the first 100 days post-transplant as described previously (3).

### 2.4 Whole exome sequencing

WES was performed at Blueprint Genetics, Helsinki, Finland. The sample preparation and exome sequencing have been previously described (22). In data analysis, we focused on 41 genes coding for components of the classical, lectin and terminal pathways as well as for complement receptors and membrane-bound complement regulators. The list of studied genes is presented in the **Supplementary Table S1**.

### 2.5 Bioinformatics analysis

The germline variant analysis was performed using BasePlayer (v.1.0.2) (23). Raw variant calls were filtered to include only good quality variants in the set (1000 genomes mapability track) (24) and quality measures GQ>=20, QUAL>=20, coverage>6 reads, and AF>=30%). Only non-synonymous and splice variants in protein-coding transcripts with a minor allele frequency (MAF) of < 0.01 in population-specific controls gnomAD (v.2.1) non-cancer whole database and gnomAD non-cancer Finns (n=10 816) (25) were considered in the study. Variants were classified as pathogenic (P), likely pathogenic(LP), benign (B), likely benign (LB) or variants of uncertain significance (VUS) according to the American College of Medical Genetics and Genomics (ACMG) (26) guidelines using InterVar (27). Also ClinVar (version 23.01.2021) was used for the variant pathogenicity estimation (28).

Findings were validated visually with BasePlayer. Locations of amino acid variants in protein structure and potential effects on protein function were analyzed by UniProt (https://www.uniprot.org) with RCSB PDB (RCSB.org) (29) data models 5FMW (30) and 7AKK (31) and drawn with Biorender (https://www.biorender.com/).

### 2.6 Statistical analysis

To study the enrichment of rare variants in the genes studied, the frequency of variants in the patient set and among gnomAD non-cancer Finns were compared. The significances of differences were evaluated using 2-sided Fisher’s exact test. Burden test of variants was done using the Total Frequency Test (TFT) (32) by collapsing rare germline variants to the gene and pathway level. These were compared to the total allele counts of variants in gnomAD non-cancer Finns with the same filtering as in the patient set. Benjamini-Hochberg procedure was used to adjust the p-values for multiple testing. The analyses were performed in R (version 4.1.2, https://www.r-project.org/).

## 3 Results

The patient data and clinical findings of our patient cohort have been published before (3). In brief, the original patient cohort consisted of 122 patients with an allogeneic HSCT between 2001 and 2013 at the Helsinki University Children´s Hospital, Finland. We evaluated the acute adverse events emerging ≤ 100 days post-transplant and prevalence of key vascular complications. We identified a total of 19/122 (15.6%) patients with vascular complications, fulfilling the criteria of capillary leak syndrome (CLS), veno-occlusive disease/sinusoidal obstruction syndrome (VOD/SOS) or thrombotic microangiopathy (TMA). These patients had a poorer 5-year overall survival than those without vascular complications (26% versus 77%, p<0.001). These endotheliopathy-related adverse events appeared early on post-HSCT, varied in their clinical phenotype and predicted a poor outcome.

### 3.1 Gene analysis

Samples for genetic analyses were available from 109 patients, of whom 17 had endotheliopathy. Of the 109 patients 50 (45.9%) had one or several, nonsynonymous, rare (MAF <0.01) germline variants. These 109 patients had altogether 51 different and in total 66 variants in 25 complement genes of soluble classical, lectin or terminal pathway factors or in the membrane-bound components (receptors or regulators). As many as 21/66 (31.8%) of the variants were in the terminal pathway. The list of gene variants is presented in **Supplementary Table S2**.

**Figure 1:**
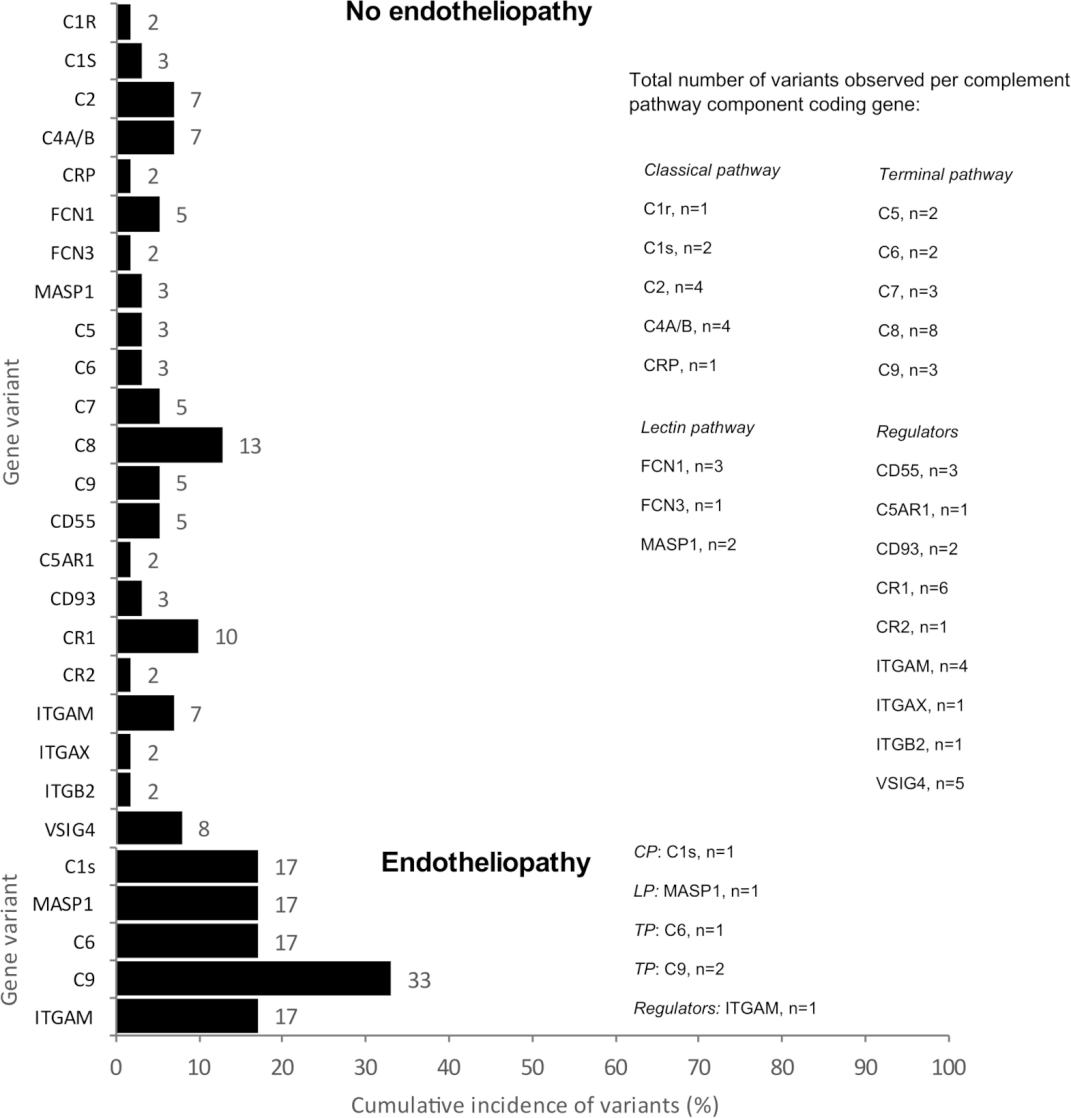
The cumulative incidence (%) and the total number (n) of variants observed per complement pathway component coding gene. The number of variants per gene is equal to the number of patients with a variant in that gene. Above are shown the variants of the patients without endotheliopathy (patient n=92) and below those with endotheliopathy (patient n=17). C1r, complement component C1r subcomponent; C1s, complement component C1s subcomponent; C2, complement component C2; C4A/B, complement components C4A and C4B; CRP, C-reactive protein; FCN1, Ficolin-1 (M-ficolin); FCN3, Ficolin-3; MASP1, Mannan-binding lectin serine protease 1; C5, complement component C5; C6, complement component C6; C7, complement component C7; C8, complement component C8; C9, complement component C9; CD55, DAF, Decay-accelerating factor; C5AR1, C5a anaphylatoxin chemotactic receptor 1; CD93, complement component C1q receptor; CR1, complement receptor type 1; CR2, complement receptor type 2; ITGAM, integrin subunit alpha M; complement receptor type 3, ITGAX, integrin subunit alpha X; ITGB2, integrin beta-2; VSIG4, V-set and immunoglobulin domain-containing protein 4 (also called complement and Ig receptor/CRIg).

**Figure 2:**
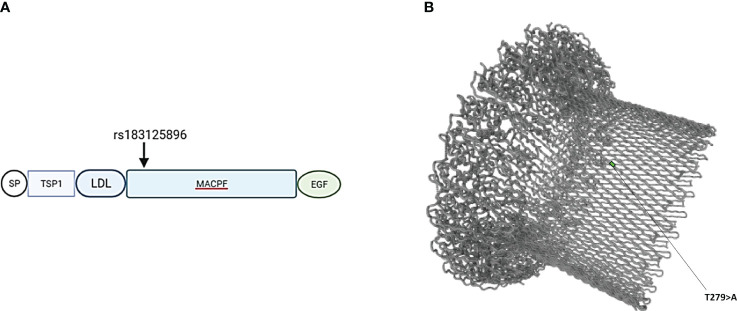
**(A)** Schematic location of variant rs183125896 encoded amino acid (Thr279Ala) in the domain structure of C9 protein. Variant is located in exon 6, which codes for the MACPF (membrane attack complex/perforin) domain. **(B)** Barrel structure of the membrane attack complex (https://www.uniprot.org). TSP1, thrombospondin type-1 repeat; LDL, low-density lipoprotein receptor class A repeat; EGF, epidermal growth factor- like domain.

**Figure 3:**
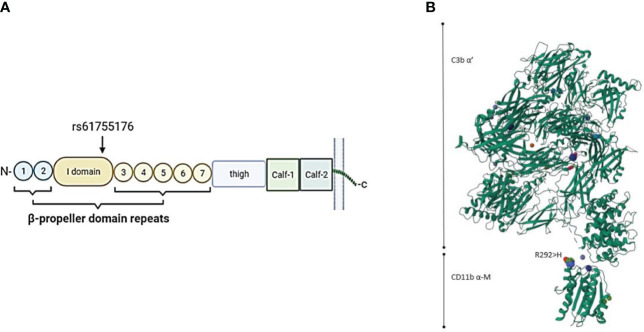
**(A)** Schematic location of the ITGAM variant rs61755176 encoded amino acid (Arg292His) in the CR3 alpha-chain (CD11b). The variant is located in exon 9 coding the edge of the I-domain surrounded by the beta-propeller domain repeat domains in the CD11b part (coded by ITGAM) of the CR3 receptor. **(B)** 3-dimensional structure of the CD11b-iC3b complex (https://www.uniprot.org). The variant amino acid is located close to the binding site of CR3 to iC3b.

Patients with endotheliopathy had variants in several complement pathways. The most notable ones were detected in the terminal pathway (C6 and C9), lectin pathway (MASP1) and in ITGAM encoding for CD11b, a part (the alpha-chain) of the β-integrin complement receptor type 3, CR3 (**Figure 1**). Examples of discovered variants are given in **Figures 2**, **3**, where the locations of the variant in protein domain sequences (2A and 3A) and structures (2B and 3B) are indicated. Four patients had the same rare (gnomAD Finn MAF= 0.009608) missense variant (rs183125896; Thr279Ala) in the C9 gene. Two of these patients were diagnosed with endotheliopathy, CLS followed by thrombotic microangiopathy (TMA), and furthermore one with a capillary leak syndrome-like (CLS-like) phenotype. One of the patients with CLS and TMA had only this C9 variant, the other also a variant in C1s (rs149869489; Pro428Arg). This C9 variant has no previously known disease associations and is classified by the ACMG guidelines as a variant of uncertain significance (VUS). A schematic C9 protein structure and the location of the variant in both C9 and the MAC complex is shown in **Figure 2**. Two patients had a rare (gnomAD Finn MAF= 0.009841) variant (rs142896559; Gly452Glu) in the C6 gene. One of these patients was diagnosed with CLS, severe anal pain and diarrhea. This C6 variant is classified as likely benign.

We found four different integrin alpha-M (ITGAM) variants in five patients, all classified as VUS. One of our patients with a missense variant in the ITGAM gene (rs61755176; Arg292His: gnomAD Finn MAF= 0.0005198) was diagnosed with CLS and TMA, but also showing signs of VOD. The other patient with a missense variant in the ITGAM gene (rs775141495; Ile13Ser: gnomAD Finn MAF<0.0001) had a similar gastrointestinal bleeding and bilirubin increase suggesting endotheliopathy, but not fulfilling the diagnostic criteria for TMA or VOD. This patient also developed hypertension and acute respiratory distress syndrome (ARDS) and died D+65 post-transplant. No infection etiology was found for the patient´s increasing oxygen requirement and pulmonary problems. The schematic structure of the α-M domain of the CR3 protein in complex with C3b and the location of variant rs61755176 on the CR3 is shown in **Figure 3**. According to this structure, the location of Arg292His is close to the attachment site to iC3b. On the other hand, and although the published protein structure of CR3 includes the integrin α-M-chain, it does not include the Ile13Ser variant (rs775141495) located in the first blade of the β-propeller domain of CD11b. Thus, no predictions can be made to its expected effect on CR3 function.

One patient with severe CLS and TMA/VOD with rapid weight gain, ascites, pleural effusion, elevated bilirubin, hepatomegaly, low levels of coagulation factors and GI-bleeding, had an ultrarare missense variant in the MASP1 gene (rs1712360640; Ala544Thr). This variant was absent in gnomAD but found in ALFA project Europe with an allele frequency <0.0001 and is classified as VUS. The variant has not been reported in ClinVar. This variant is located in the serine protease domain, but its possible effect on the protease function is not known.

**Figure 4:**
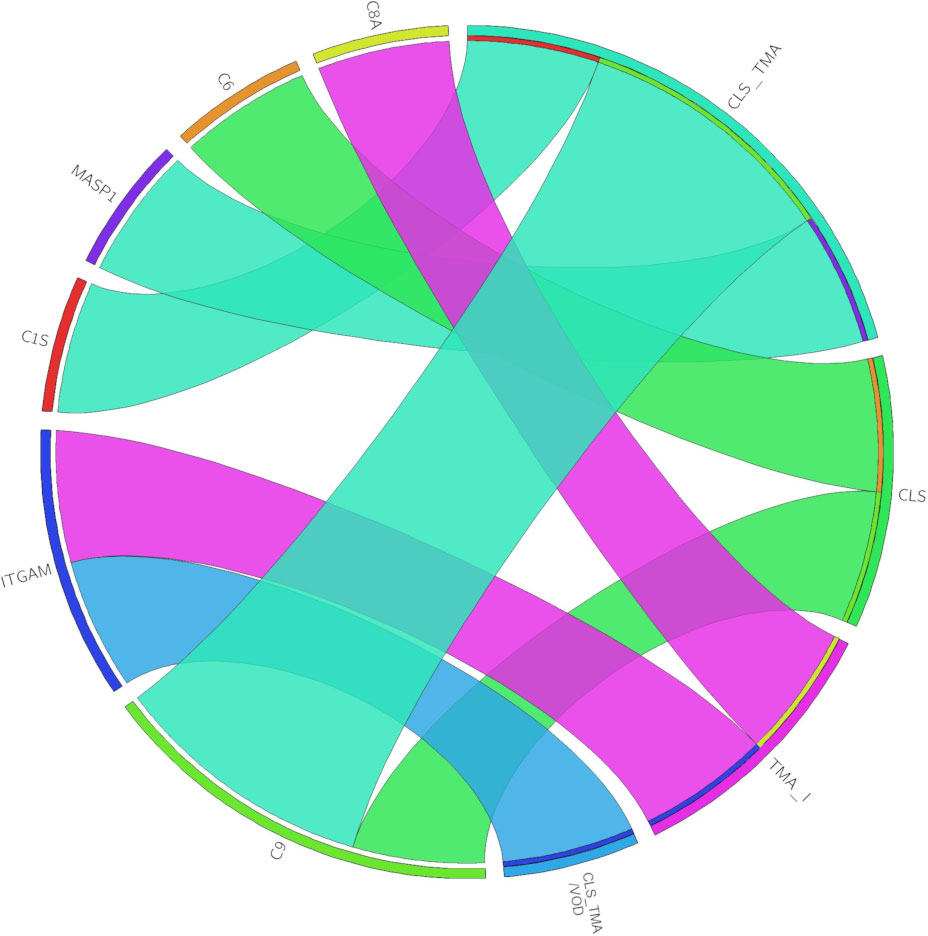
The chord diagram depicts the associations between genetic findings (left) and diagnoses (right). The width of each chord is determined by the number of patients in the diagnosis group with variants in the gene. CLS, capillary leak syndrome; TMA, thrombotic microangiopathy; TMA_I, TMA-like; CLS_TMA, CLS+TMA; CLS_TMA/VOD, CLS+TMA/VOD.

Compared to gnomAD, the gene variants seen in our patients with endotheliopathy were all significantly (FDR < 0.05) enriched. The associations between the genetic findings and endotheliopathy diagnoses are shown in **Figure 4**. Overall, 14/25 genes coding for components of the complement system had an increased burden of missense variants among our patients in comparison to the gnomAD Finnish population. In our pathway burden analysis, variants of the classical pathway of complement activation were found to be enriched in the patients in comparison to gnomAD controls. All complement gene variants enriched in our patient population are shown in **Table 2**. Although a fair comparison can be made only to the gnomAD Finnish population, we also made a comparison to gnomAD Europe (**Supplementary Table S2**). It showed features that distinguish the Finnish population from the rest of Europeans.

**Table 2:** Complement gene variants enriched in the patient population (N=109) compared to gnomAD non-cancer Finn population (N=10 816).

	Variants in cohort (N)	Variants in gnomad (N)	p-value	q-value	OR	CI95
*Classical pathway*	14	668	0.0131		2.08	1.21-Inf
C1S	3	19	0.0014	0.0035	15.65	3.89-Inf
C2	4	26	0.0002	0.0009	15.25	4.80-Inf
C4B	4	3	0.0000	0.0000	131.35	28.38-Inf
*Lectin pathway*	7	703	ns			
FCN1	3	16	0.0009	0.0025	18.58	4.55-Inf
MASP1	3	38	0.0083	0.0160	7.83	2.02-Inf
*Terminal pathway*	20	1594	ns			
C6	3	34	0.0063	0.0131	8.75	2.25-Inf
C7	3	25	0.0028	0.0064	11.90	3.01-Inf
C8A	6	33	0.0000	0.0000	18.02	7.23-Inf
C9	5	22	0.0000	0.0001	22.52	8.01-Inf
*Regulators and receptors*	24	2253	ns			
CD55	3	13	0.0005	0.0017	22.87	5.49-Inf
CD93	2	17	0.0156	0.0279	11.66	1.90-Inf
CR1	6	44	0.0000	0.0001	13.51	5.50-Inf
ITGAM	5	29	0.0000	0.0001	17.09	6.20-Inf
VSIG4	4	14	0.0000	0.0001	28.28	8.45-Inf

C1s, complement component C1 subcomponent s; C2, complement component 2; C4B, complement component C4B; FCN1, Ficolin-1 (M-ficolin); MASP1, Mannan-binding lectin serine protease 1; C6, complement component C6; C7, complement component C7; C8A, complement component C8 alpha subunit; C9, complement component C9; CD55, DAF, Decay-accelerating factor; CD93, complement C1q receptor; CR1, complement receptor type 1; ITGAM, integrin subunit alpha M; complement receptor 3, VSIG4, V-set and immunoglobulin domain containing 4 (also called complement and Ig receptor (CRIg).

The first row in each category indicates the complement pathway burden association of all the rare variants observed in this pathway. Also Benjamini-Hochberg method corrected p-values (q-value) are given. ns, not significant.

## 4 Discussion

The present study found a higher-than-expected frequency of rare complement gene variants possibly related to vascular complications following HSCT in children. Particularly interesting associations were found to the terminal pathway complement components C6 and C9 as well as to the main phagocytic complement receptor type 3 (CR3, CD11b/CD18). While pathway-burden analysis indicated that rare missense variants in genes coding for classical pathway are enriched in patients in general, from the point of view of endotheliopathy, main links were found to the terminal pathway complement components.

We found five patients with mutations in the ITGAM gene encoding for CD11b (integrin alpha-M), a central part of the key complement receptor CR3. CD11b alpha chain forms the CR3 receptor together with the integrin beta 2 chain (CD18 encoded by ITGB2). CR3 binds structures coated with iC3b, which is an inactivated form of C3b. CR3 regulates the adherence of neutrophils and monocytes to stimulated endothelium. Importantly, CR3 mediates the phagocytosis of complement iC3b-coated microbes and other particles. CR3 functions also as a receptor for the coagulation system proteins fibrinogen and factor X (FX). Mutated ITGAM may thus result in diminished neutrophil adhesion and migration, reduced phagocytosis, increased cytokine production and elevated anti-dsDNA antibody formation, which may further trigger the complement cascade (33). Genetic variants in the ITGAM gene are strongly associated with systemic lupus erythematosus (SLE) and its complications (lupus nephritis, etc) (33), as well as with pre-eclampsia (34). CR3 and CR4 are both β2-integrins that specifically recognize iC3b. It has been previously found that variant rs2230424 in CR4, located in the first blade of the β-propeller domain, results in a decreased binding capacity of CR4 to iC3b (34). We speculate, that ITGAM variant (rs775141495), also in the first blade of the β-propeller domain, may also interfere with the binding of CR3 to iC3b.

Acute respiratory distress syndrome (ARDS) is characterized by an increased permeability of endothelium and epithelium leading to an inflammatory reaction with accumulation of neutrophils and a protein-rich edema of the lungs. Later, also macrophages, strongly expressing CR3, promote the inflammatory response. Biomarkers of the endothelial damage have been linked to increased morbidity and mortality (35–37). One of our patients with the ITGAM variant (rs775141495; Arg292His) had an endotheliopathy-like clinical phenotype with ARDS. The patient´s ARDS symptoms evolved from day 30 post-transplant, thus matching the timeline of the post-engraftment acute phase (30-100 days post-transplant) pulmonary complications. An impaired cellular immunity plays a central role in this phase in both the infectious and non-infectious complications with the latter including many entities related to endothelial damage (38, 39). Our patient with the ITGAM variant Arg292His with CLS followed by TMA/VOD was treated with defibrotide (6.25mg/kg iv q. 6 hours) and survived. The other one with ARDS was not treated with defibrotide and died. Defibrotide is found to diminish the endothelial activation and enhance the local antithrombotic effects, while having also anti-inflammatory effects by interacting with the endothelium (40). In the clinical trial by Frame et al. (41) defibrotide was found safe, and even with the study not being designed to address the efficacy of defibrotide, the survival rates of the patients treated with defibrotide were promising, when compared to other patients with SARS-CoV-2 ARDS.

Mannan-binding lectin-associated serine protease -1 (MASP-1) is an initial part of the lectin pathway and can promote activation of both complement and coagulation. It is a procoagulant by inducing fibrin formation with thrombin, but it also directly activates prothrombin, FXIII and thrombin-activatable fibrinolysis inhibitor (TAFI) (42, 43). Thrombin, on the other hand, has been reported to directly activate the complement cascade by cleaving C3 and C5 (44). The lectin pathway was suggested to mediate complement activation in SARS-CoV-2 infection, and the MASP2-inhibitor narsoplimab had a promising effect in critically ill COVID-19 patients (45). The lectin pathway may also play a role in HSCT-related TMA. Adult patients with HSCT-TMA treated in a phase II study with narsoplimab showed significant improvement in TMA-related laboratory values, and the treatment resulted in a good clinical response with improved overall survival (46). Our patient with the MASP-1 variant Ala544Thr had severe and persistent diffuse gastrointestinal bleeding leading to ICU-admission twice and ending up being lethal. Massive haemorrhage is thought to be a possible trigger of complement activation (47, 48) and activated complement could contribute to tissue damage seen at the site of the haemorrhage (49, 50). We speculate that the MASP-1 variant rs1712360640 with severe GI-bleeding could lead to a more vigorous activation of the complement and coagulation cascade, leading to endothelium-related complications.

C6 is an essential part of the membrane attack complex (MAC). The variant (rs142896559; Gly452Glu) seen in our patient with CLS in located in the MAC perforin (MACPF) domain of the C6 protein (51). The variants seen in C6 and C9 are both located in the MACPF domain. The functional consequences of this variant in C6 are currently not known. However, the essential role of C6 in the generation of the complement membrane attack complex (MAC) and location of the variant in one of the key domains suggests that it could be involved in abnormal assembly of MAC.

While the functional consequences of the C9 variant (rs183125896; Thr279Ala) observed in four patients are also unclear, interestingly, reports on other C9 variants in connection to TMA have recently been published (52, 53). It is therefore possible that, in the HSCT patients, this mutation, normally tolerable, could contribute to triggering TMA.

Many variants were overrepresented in the whole HSCT population (**Table 2**). Interestingly, also transplant patients without vascular problems showed enrichment of variants in many complement genes. This could be related to the underlying reasons for the HSCT (usually malignancy, but also hematopoietic syndromes, immunological or metabolic disorders; **Table 1**). In this scenario, complement may have functioned insufficiently to prevent the disorder. Furthermore, the variants observed in the endotheliopathy patients were enriched in the patient population, although the small sample size of endotheliopathy patients did not warrant direct statistical comparison. A reason for this could be an incompatibility in the complement system between the recipient and the donor. Components from the donor and the recipient (e.g. between an activating and a regulating factor) may have a mismatch with each other and lead to events that harm the vascular endothelium. This hypothesis is supported by the fact that patients with endotheliopathy had more matched unrelated donors than those without endotheliopathy (p=0.031).

The limitation of our study is the size of our cohort. Gene burden analysis may be influenced by the relatively small N and the findings should be evaluated using a larger validation cohort. The strength of our study is the uniform Finnish ethnicity of the patients. Because of the centralization of more than 95% of the paediatric HSCTs in Finland to one, single centre, the cohort covers the whole Finland and represents the national population well. Thus, the frequency of variants in the patient set and gnomAD non-cancer Finns was comparable. Comparison to gnomAD Europeans (**Supplementary Table S2**) showed differences in complement variant distribution between Finns and the rest of Europeans. This is understandable, because it is well known that Finns are a genetically relatively uniform but distinct population in Europe (54).

Treatment-related toxicity is still a significant clinical problem in paediatric allo-HSCT. Injury to the vascular endothelium is relatively common after HSCT with varying phenotypic appearances suggesting hitherto unidentified underlying mechanisms. Rare and ultrarare variants in complement genes are overexpressed in patients with injury to the vascular endothelium. These nonsynonymous variants in complement genes may be related to endotheliopathy and poor prognosis seen in these patients. Our hypothesis is that in addition to these variants, also genetic incompatibility between the donor and the recipient predisposes to endothelial damage and problems seen post-HSCT. Studies to address this hypothesis are underway.

## Data availability statement

The datasets presented in this article are not readily available because of ethical and privacy restrictions. Requests to access the datasets should be directed to the corresponding author/s.

## Ethics statement

The studies involving humans were approved by The Research Ethics Committee of the Helsinki University Hospital. The studies were conducted in accordance with the local legislation and institutional requirements. The ethics committee/institutional review board waived the requirement of written informed consent for participation from the participants or the participants’ legal guardians/next of kin because all samples and data were obtained following authorization by the ethics committee and the national supervisory authority Valvira. Written, informed consents were not attainable due to the retrospective nature of the study also including deceased patients.

## Author contributions

LL performed research and collected data; JK analyzed and interpreted data; KV and OK supervised the students and interpreted data; IL and SM designed research, supervised the students, and interpreted data. LL, JK, KV, IL, and SM wrote the manuscript. All authors contributed to the article and approved the submitted version.
