# Supplementary material for: Rare variants in complement system genes associate with endothelial damage after pediatric allogeneic hematopoietic stem cell transplantation
Source: Front Immunol. 2023 Sep 13;14:1249958. doi: 10.3389/fimmu.2023.1249958 (PMC10525714; doi:10.3389/fimmu.2023.1249958)
Supplement: Supplementary file 1 [file DataSheet_1.pdf]

Supplementary Table S1. The 41 complement genes sequenced from our 109 pediatric patients having received allo-HSCT at the Helsinki University Children´s Hospital during 2001-2013.

PATHWAY	GENE	PROTEIN
Classical pathway	C1QA	C1q A Chain
	C1QB	C1q, Complement component 1, q subcomponent, beta polypeptide<br>complement component C1q, B chain
	C1QC	C1qc; Complement component 1, q subcomponent, C chain
	C1R	C1r, Complement component 1, r subcomponent
	C1S	C1s, Complement component 1, s subcomponent
	C2	C2
	C4A	C4A
	C4B	C4B
	CRP	CRP
	PTX3	PTX3: Pentraxin 3, long
Lectin pathway	MBL	MBL, Mannan binding lectin
	MASP1	MASP-1
	MASP2	MASP-2
	FCN1	Ficolin-1 (M-ficolin)
	FCN2	Ficolin-2 (L-ficolin)
	FCN3	Ficolin-3 (H-ficolin)
Terminal pathway	C5	C5, Complement component 5
	C6	C6, Complement component 6
	C7	C7, Complement component 7
	C8A	C8A, Complement component 8, Alpha subunit
	C8B	C8B, Complement component 8, Beta subunit
	C8G	C8G, Complement component 8, Gamma subunit
	C9	C9, Complement component 9
Membrane bound regulators	CD59	CD59, Protectin, human leukocyte antigen MIC11)
	CR1	CR-1, Complement receptor 1
	VSIG4	CRIg, Immunoglobulin superfamily protein Z39IG; Z39IG<br>complement receptor of the immunoglobulin superfamily; CRIG
	CD55	DAF, Decay-accelerating factor for complement
	CD46	MCP, Membrane cofactor protein
	C5AR1	C5AR, Complement component 5 receptor 1; C5R1, C5a anaphylatoxin receptor; C5AR<br>CD88 antigen; CD88)
	C5AR2	C5L2 (G Protein-coupled receptor 77; GPR77)
	C3AR1	C3AR, Complement component 3a receptor 1; C3AR1
	CD93	C1QR, Complement component 1, q subcomponent, receptor 1; C1QR1, D93 antigen; CD93<br>complement component 1q receptor; C1QR<br>collectin receptor
	C1QBP	gC1qR, C1q globular domain-binding protein (complement component C1q-binding protein; C1QBP,<br>hyaluronic acid-binding protein 1; HABP1<br>P32 splicing factor SF2-associated protein)
	CR2	CR2, CD21
	ITGAM	CR3, CD11b
	ITGAX	CR4, CD11c
	ITGB2	CD18, Integrin beta 2
	CALR	CALR, Calreticulin
Adiponectin	ADIPOQ	Adiponectin, adipocyte-, C1q-, and collagen domain-containing; ADIPOQ
	ADIPOR1	Adiponectin receptor 1; ADIPOR1
	ADIPOR2	Adiponectin receptor 2; ADIPOR2
