# Supplementary material for: Rare variants in complement system genes associate with endothelial damage after pediatric allogeneic hematopoietic stem cell transplantation
Source: Front Immunol. 2023 Sep 13;14:1249958. doi: 10.3389/fimmu.2023.1249958 (PMC10525714; doi:10.3389/fimmu.2023.1249958)
Supplement: Supplementary file 2 [file DataSheet_2.pdf]

Supplementary Table S2. The gene variants in the classical, lectin and terminal pathways and membrane-bound regulators of the 109 pediatric patients having received allo-HSCT at the Helsinki University Children´s Hospital during 2001-2013.

Gene	rs-ID	Patients (n)	Mutation	Amino acid change	gnomAD Fin MAF	gnomAD Eur MAF	ClinVar	Intervar	SIFT	SIFT score[1]	PolyPhen	Probability score[2]
Classical pathway												
C1R	rs771519326	1	C>T	V403M	0.00004068	0.0001319	UNK	VUS	tolerated	UNK	benign	UNK
C1S	rs149869489	2	C>G	P428R	0.002906	0.0003882	UNK	VUS	deleterious	0	benign	0.248
C1S	novel	1	A>T	N642Y	N/A	N/A	N/A	N/A	N/A	N/A	N/A	N/A
C2	rs9332736	2	del (TGGTGGACAGGGTCAGGAATCAGGAGTC)	frameshift NA ⇒ P [Pro] stopgain	0.005937	0.007511	Pathogenic/likely pathogenic	VUS	N/A	N/A	N/A	N/A
C2	rs769958879	1	G>A	E394K	0.0001387	0.000	UNK	VUS	tolerated	0.15	benign	0.001
C2	rs144201432	1	A>G	N64S	0.00003983	0.0007292	VUS	VUS	tolerated	0.26	benign	0
C4A	rs557313902	1	C>T	A891V	0.0002877	0.000	UNK	VUS	deleterious	0.01	probably damaging	0.983
C4B	rs1387676878	1	C>T	Q1166*	N/A	N/A	U NK	VUS	N/A		N/A	
C4B	rs2229409	1	G>T	R1210L	0.000	0.00008994	UNK	VUS	tolerated	0.22	benign	0.132
C4B	rs2229403	1	C>T	A1207V	0.00003263	0.000	UNK	VUS	tolerated	0.06	benign	0
CRP	rs77832441	1	G>A	T59M	0.0004036	0.002523	UNK	VUS	deleterious	0.01	probably damaging	0.998
Lectin pathway												
FCN1	rs148649884	2	C>T	A218T	0.001155	0.0008051	UNK	VUS	deleterious	0	probably damaging	0.984
FCN1	rs147539232	1	G>A	T150M	0.009130	0.006183	UNK	VUS	deleterious	0.04	probably damaging	0.88
FCN3	rs770650851	1	- > T	R288RX	0.001235	0.0001551	UNK	VUS	N/A		N/A	
MASP1	rs199628370	1	C>T	G103S	0.006374	0.0001707	UNK	VUS	deleterious	0.02	benign	0.393
MASP1	rs768013314	1	G>T	R56S	0.0003193	0.00002327	UNK	VUS	deleterious	0.01	probably damaging	0.949
MASP1	rs1712360640	1	C>T	A554T	N/A	0.0 (ALFA project Europe)	UNK	VUS	N/A	N/A	N/A	N/A
Terminal pathway												
C5	rs147430470	1	A>G	I330T	0.0009123	0.001520	Likely benign	Likely benign	tolerated	0.32	benign	0
C5	rs200674959	1	T>C	I1291V	0.0007165	0.0004799	VUS	VUS	tolerated	0.28	benign	0.073
C6	rs142896559	2	C>T	G452E	0.009841	0.006520	Likely benign	Likely benign	deleterious	0	benign	0.01
C6	rs41271067	1	T>C	D696G	0.005096	0.01185	Benign	Likely benign	tolerated	0.16	benign	0.062
C7	rs121964920	1	C>A	R521S	0.0007621	0.004135	Pathogenic	VUS	deleterious	0	probably damaging	0.964
C7	rs200737768	1	G>A	A670T	0.0002405	0.001023	UNK	VUS	tolerated	0.79	benign	0.001
C7	rs200879856	1	G>A	V536I	0.00004021	0.0001898	UNK	VUS	tolerated	1	benign	0
C8A	rs41285938	3	C>T	P575S	0.003902	0.01459	UNK	VUS	tolerated	0.25	benign	0.007
C8A	rs150404785	2	G>A	D129N	0.002269	0.003528	VUS	VUS	tolerated	1	benign	0.049

C8A	rs143908758	1	G>A	R444H	0.004816	0.005240	Likely benign	VUS	deleterious	0.01	probably damaging	0.985
C8B	rs139498867	1	C>A	D382Y	0.002946	0.006120	Likely benign	Likely benign	deleterious	0	possibly damaging	0.847
C8G	rs143480441	1	C>G	P23A	0.0002874	0.0002702	UNK	VUS	tolerated	1	benign	0
C9	rs183125896	4	T>C	T279A	0.009608	0.0003976	UNK	VUS	tolerated	0.19	benign	0.22
C9	rs142654382	1	A>G	Splice Donor Variant	0.004699	0.0001245	UNK	VUS	N/A	N/A	N/A	N/A
Regulators and receptors												
CD55	rs147474393	1	T>G	L82R	0.004180	0.00004648	UNK	Likely benign	deleterious	0.05	possibly damaging	0.863
CD55	rs60822373	1	G>T	A163S	0.007821	0.0001244	UNK	VUS	tolerated	0.14	probably damaging	0.976
CD55	Novel	1	C>G	S370C	N/A	N/A	N/A	N/A	N/A	N/A	N/A	N/A
C5AR1	rs201037165	1	C>T	A88V	0.0008777	0.0002170	UNK	VUS	tolerated	0.08	possibly damaging	0.464
CD93	rs202080643	1	C>T	G314E	0.0002398	0.00003150	UNK	VUS	tolerated	1	benign	0
CD93	rs144703062	1	C>T	V416I	0.003188	0.008997	UNK	VUS	tolerated	0.26	benign	0.013
CR1	rs200076672	3	C>T	T1929I	0.002325	0.0001572	UNK	VUS	tolerated	0.21	probably damaging	0.996
CR1	rs55998388	1	G>A	Splice Donor Variant	0.00004034	0.0006993	UNK	VUS	N/A	N/A	N/A	N/A
CR1	rs746473308	1	C>T	R758C	0.0005357	0.000008978	UNK	VUS	deleterious	0.02	possibly damaging	0.738
CR1	rs761231049	1	C>T	R919C	0.0001199	0.00004685	UNK	VUS	deleterious	0.02	probably damaging	0.992
CR2	rs140808707	1	G>A	R826H	0.003441	0.0008025	Likely benign	Likely benign	tolerated	1	benign	0.003
ITGAM	rs184282943	2	C>T	R197W	0.003677	0.0002725	UNK	VUS	tolerated	0.24	benign	0.05
ITGAM	rs61755176	1	G>A	R292H	0.0005198	0.001091	UNK	VUS	tolerated	0.02	benign	0
ITGAM	rs1385680743	1	A>T	D641V	0.00004641	0.000	UNK	VUS	deleterious	0.01	benign	0.169
ITGAM	rs775141495	1	T>G	I13S	0.000	0.00004424	UNK	VUS	tolerated	1	benign	0.237
ITGAX	rs17853815	1	G>A	E547K	0.002031	0.003417	UNK	VUS	deleterious	0.03	benign	0.327
ITGB2	rs138303556	1	C>T	G69R	0.000	0.000008998	Conflicting interpretations of pathogenicity	VUS	N/A	N/A	N/A	N/A
VSIG4	rs41307375	2	G>A	T289I	0.008753	0.008693	Benign	Likely benign	deleterious	0	benign	0.262
VSIG4	rs41306131	2	C>A	V92F	0.008697	0.008894	Benign	Likely benign	deleterious	0.03	possibly damaging	0.478
VSIG4	rs199904204	1	C>T	G11R	0.006667	0.0003464	UNK	VUS	tolerated	0.07	benign	0.044

1 SIFT score: 0.0-0.05 deleterious, 0.05-1.0 tolerated. 2 PolyPhen probability score: 0.0-0.15 benign, 0.15-1.0 possibly damaging, 0.85-1.0 probably damaging. Abbreviations: MAF= minor allele frequency, UNK=unknown, VUS=variant of uncertain significance, C1r = complement component C1r subcomponent, C1s = complement component C1s subcomponent, C2 = complement component C2, C4A/B = complement components C4A and C4B, CRP = C-reactive protein, FCN1 = Ficolin-1 (M-ficolin), FCN3 = Ficolin-3, MASP1 = Mannan-binding lectin serine protease 1, C5 = complement component C5, C6 = complement component C6, C7 = complement component C7, C8 = complement component C8 , C9 = complement component C9, CD55 = DAF, Decay-accelerating factor, C5AR1 = C5a anaphylatoxin chemotactic receptor 1, CD93 = complement component C1q receptor, CR1 = complement receptor type 1, CR2 = complement receptor type 2, ITGAM = integrin subunit alpha M, complement receptor type 3, ITGAX = integrin subunit alpha X, ITGB2 = integrin beta-2, VSIG4 = V-set and immunoglobulin domain-containing protein 4 (also called complement and Ig receptor/CRIg).
